# Leaflet fracture and embolization from an On-X mechanical mitral valve

**DOI:** 10.1016/j.xjtc.2020.06.021

**Published:** 2020-06-24

**Authors:** Erik Helgeland, Kristoffer Andresen, Karl Andreas Dumont, Johannes Lagethon Bjørnstad

**Affiliations:** a Department of Cardiothoracic Surgery, Oslo University Hospital, Rikshospitalet, Oslo, Norway; b Department of Cardiology, Oslo University Hospital, Rikshospitalet, Oslo, Norway; c Institute of Clinical Medicine, University of Oslo, Oslo, Norway

**Figure:**
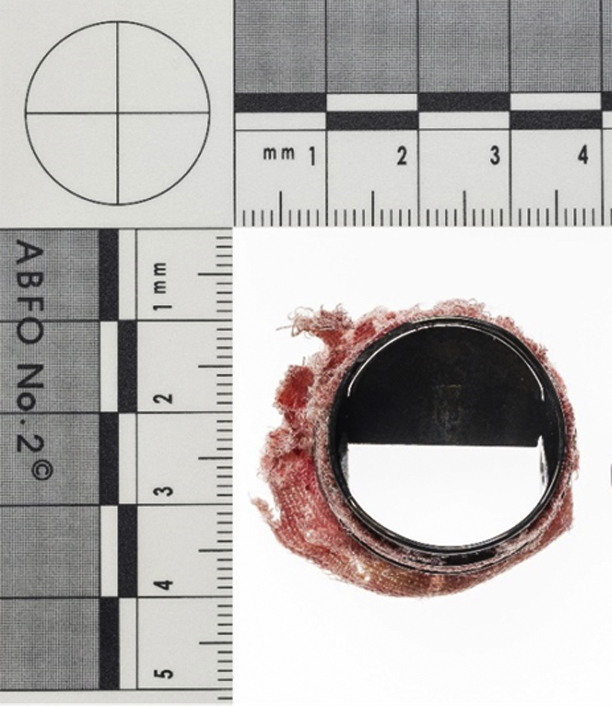
Explanted On-X mitral prosthesis with 1 leaflet missing.

**Central Message**

We report the case of a 26-year-old male who presented with cardiogenic shock due to leaflet fracture and embolization from an On-X mitral valve prosthesis that had been implanted just 1 year earlier.

See Commentary on page 144.

Leaflet escape is a dangerous but exceedingly rare complication of modern mechanical heart valves. Whereas some older models (in particular, a version of the Björk–Shiley tilting disc prosthetic valve) reported a 3.9% cumulative incidence of leaflet escape,^1^ modern mechanical bileaflet heart valves rarely fail. Only 3 previous cases of leaflet embolization from On-X mechanical valves have been reported, 2 in a mitral position and 1 in an aortic position.^2^, ^3^, ^4^ Here we report the fourth case, involving only the second patient to survive and recover from surgery.

## Case Description

A 26-year-old man presented to the emergency department of his local hospital with acute onset of dyspnea during treadmill exercise. One year earlier, he had undergone elective mitral valve replacement at another institution with an On-X 25/33 mitral valve with conform sewing ring (Cryolife, Kennesaw, Ga) to treat rheumatic mitral stenosis with regurgitation. He was admitted in cardiogenic shock with pulmonary edema and hypotension after minor hemoptysis. Transthoracic echocardiography (TTE) revealed a large mitral regurgitation and increased antegrade velocity of 2.5 m/s over the prosthesis, which prompted suspicion of prosthetic valve thrombosis, despite an international normalized ratio of 2.3 at the time of admission. He was stabilized with noninvasive continuous positive pressure ventilation before transfer to a tertiary hospital.

**Figure 1:**
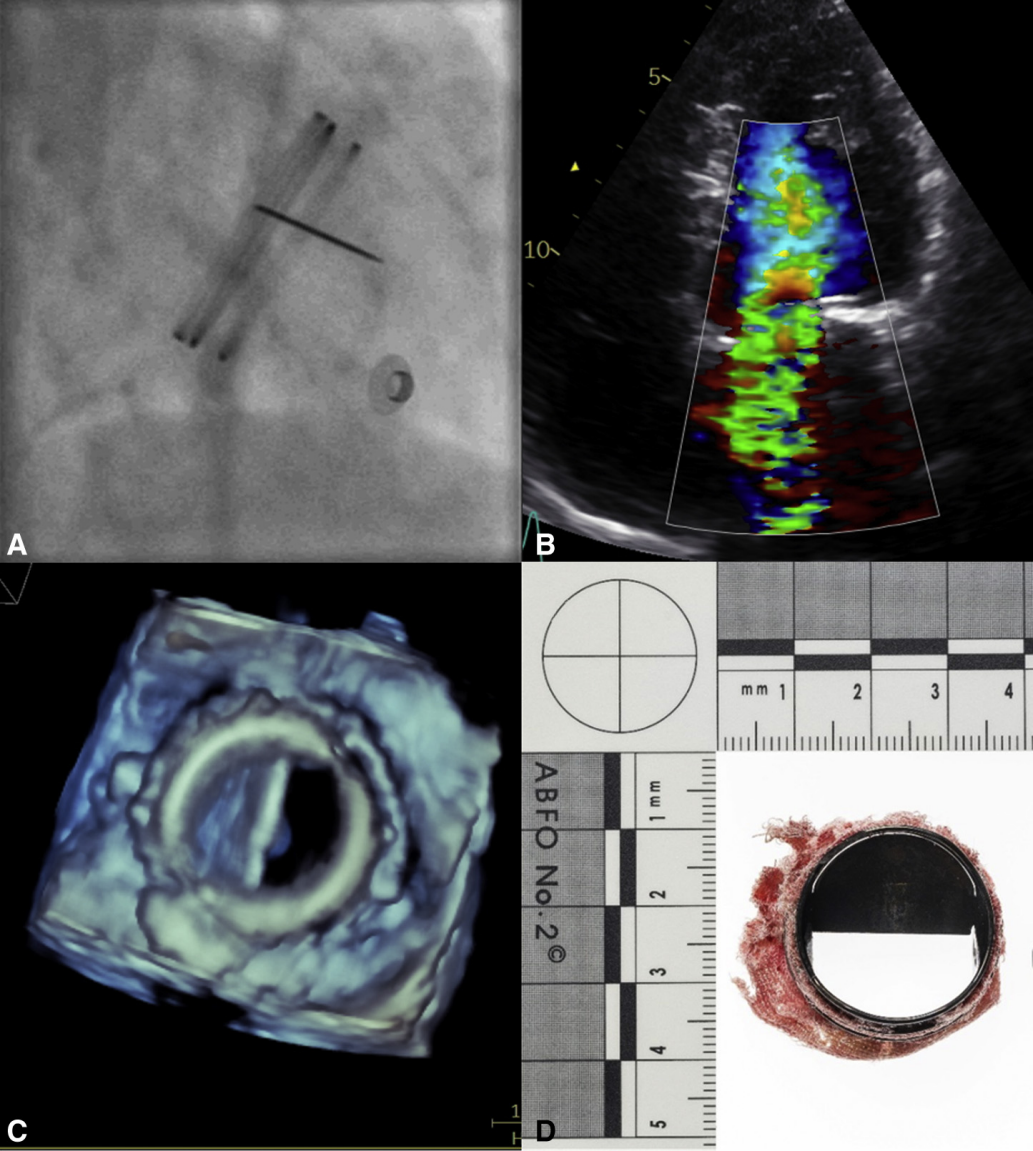
Fluoroscopy (A), transthoracic echocardiography (B), and transesophageal echocardiography with 3D reconstruction (C) of a patient in cardiogenic shock showing the mitral prosthesis with 1 missing leaflet and significant regurgitation. D, After emergency mitral valve replacement, the explanted prosthesis with 1 leaflet clearly missing.

He was taken directly from the emergency room to the cardiac catheterization laboratory, where fluoroscopy revealed a single leaflet opening (Figure 1, *A*, and Video 1) and TTE confirmed the single moving leaflet and significant mitral regurgitation in the mitral valve prosthesis (Figure 1, *B*). Intravenous thrombolysis was administered on the suspicion of acute valve thrombosis, with no beneficial or adverse effects.

(Photo credit: Øystein Horgmo, University of Oslo.)

The next morning, the patient was still in cardiogenic shock and was stabilized with an intra-aortic balloon pump. Repeat TTE raised a suspicion that the nonvisualized leaflet was not in situ, which was later supported by transesophageal echocardiography (Figure 1, *C*, and Video 1). Plain X-ray of the abdomen could not visualize any escaped leaflet. Thus, he was taken directly to the operating room for emergency mitral valve surgery, which confirmed the diagnosis of leaflet escape. The old prosthesis was explanted (Figure 1, *D*) and replaced with a new prosthesis of the same size and brand.

**Figure 2:**
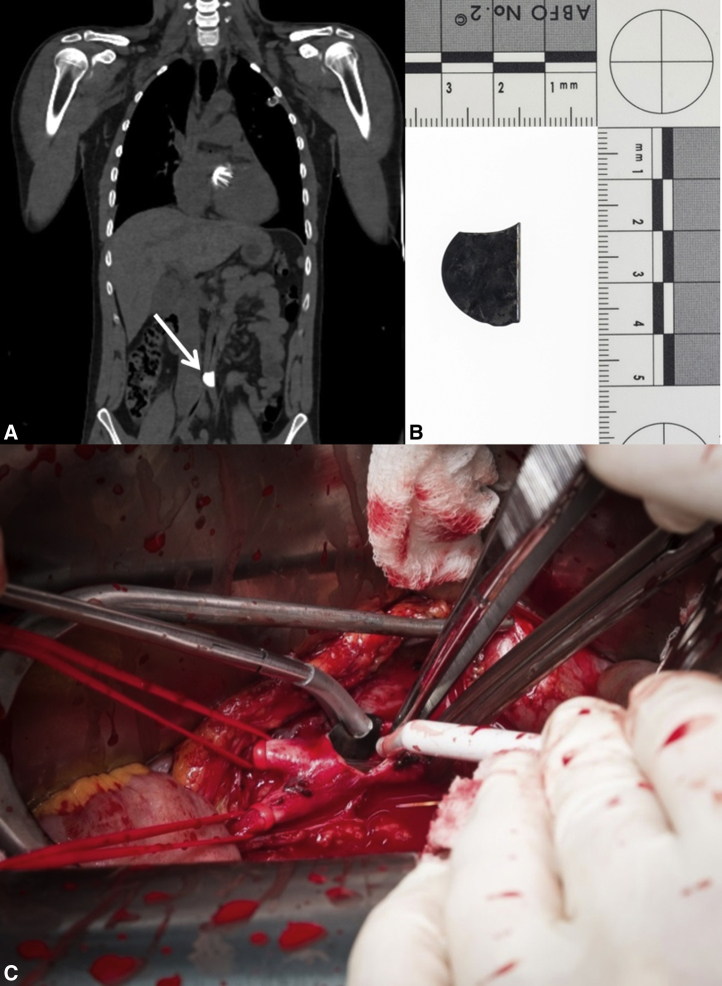
A, Postoperative computed tomography scan without contrast showing the location of the embolized leaflet in the abdominal aorta (*white arrow*). Three quarters of the missing valve lid (B) was removed from the abdominal aorta via laparotomy (C). The remaining one-quarter was not accounted for but was suspected to lay distal to the knee, the only area not imaged.

Postoperative computed tomography scan without contrast located the missing leaflet in the abdominal aorta just above the bifurcation (Figure 2, *A*). Laparotomy was performed the next day, and the valve lid was explanted through a transverse aortotomy (Figure 2, *B* and *C*). Only after vessel closure was the intra-aortic balloon pump removed under direct visual control to avoid any tears or damage to either the device or the vessel wall. The remaining one-quarter of the leaflet was not accounted for but was suspected to lay distal to the knees, the only area not yet imaged. The patient was discharged to his local hospital 4 days after admission and was doing well without any lasting disability at his 3-month postoperative follow-up. Written informed consent has been obtained for the publication of this case report.

(Photo credit: Øystein Horgmo, University of Oslo.)

## Discussion

Our institution began using the On-X valve prosthesis in 2000, and by 2019 we had implanted 775 mechanical mitral prostheses, of which 426 were On-X valves. We perform 59% of all the mitral valve operations in Norway (Norwegian Heart Surgery Registry; https://www.kvalitetsregistre.no/registers/norsk-hjertekirurgiregister.) This is the first case of leaflet fracture that we have observed in this prosthesis, and only the fourth such case published in the literature to date. One other reported failure involved the On-XMC 25/33, which has the conform sewing ring^2^; the devices used in the other 2 cases (one in the aortic position^4^ and the other in the mitral position^3^) are not known.

As illustrated in this case report, distinguishing leaflet escape from valve thrombosis can be challenging with fluoroscopy and TTE. Transesophageal echocardiography can provide valuable information when evaluating suspected prosthetic valve dysfunction but is not always available or may require anesthesia, which could destabilize a hemodynamically compromised patient. However, a noncontrast computed tomography scan is fast, readily available, and does not require sedation, and in this case, it could have made the diagnosis indirectly by localizing the escaped leaflet. Of note, we could not visualize the fragment using plain X-ray. Because this is one of the earliest leaflet escapes on record with any modern mechanical valve prosthesis, the fractured leaflet was examined by scanning electron microscopy, which could not discern any certain cause of the fracture. In its own review, the manufacturer was not able to identify any flaws or errors in the production of the prosthesis. It is well known that surgical mishandling during implantation of mechanical prosthesis can create microscopic surface scratches, leading to weak points for later fracture.

## Supplementary Data

Video 1 Fluoroscopy and transesophageal echocardiography with 3D reconstruction of a patient in cardiogenic shock with a leaflet of the On-X mitral prosthesis missing. Video available at: https://www.jtcvs.org/article/S2666-2507(20)30299-6/fulltext.
