# Supplementary figures and images for: Leaflet fracture and embolization from an On-X mechanical mitral valve
Source: JTCVS Tech. 2020 Jun 24;3:140–3. doi: 10.1016/j.xjtc.2020.06.021 (PMC8302983; doi:10.1016/j.xjtc.2020.06.021)

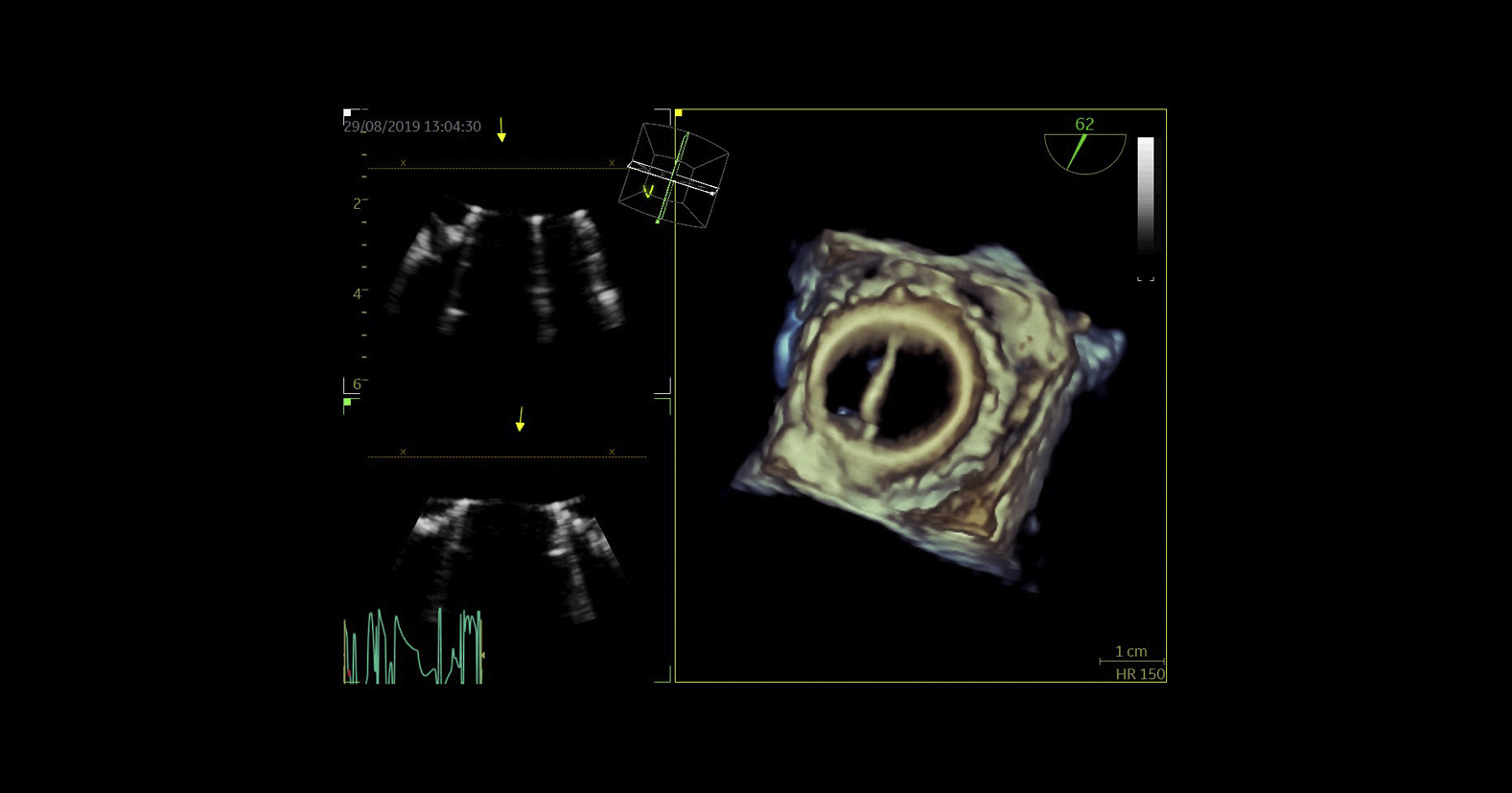

Supplement: Video 1 — Fluoroscopy and transesophageal echocardiography with 3D reconstruction of a patient in cardiogenic shock with a leaflet of the On-X mitral prosthesis missing. Video available at: https://www.jtcvs.org/article/S2666-2507(20)30299-6/fulltext. [file fx2.jpg]
